# Incidence trends and age distribution of colorectal cancer by subsite in Guangzhou, 2000–2011

**DOI:** 10.1186/s40880-015-0026-6

**Published:** 2015-08-06

**Authors:** Qin Zhou, Ke Li, Guo-Zhen Lin, Ji-Chuan Shen, Hang Dong, Yu-Ting Gu, Hua-Zhang Liu

**Affiliations:** Department of Biostatistics and Cancer Registration, Guangzhou Center for Disease Control and Prevention, Guangzhou, Guangdong 510060 P.R. China

**Keywords:** Colorectal cancer, Incidence, Trend, Subsite

## Abstract

**Introduction:**

Colorectal cancer (CRC) is the third most common cancer in China. The incidence of CRC has been increasing in recent years. The aim of this study was to explore the incidence trends and the age distribution of CRC by subsite in Guangzhou between 2000 and 2011.

**Methods:**

A total of 22,432 incident cases of CRC between 2000 and 2011 from Guangzhou Cancer Registry were identified. Crude incidence and age-standardized rates (ASRs), using the Segi’s world standard population, were calculated for CRC and CRC subsites. The incidence trend was analyzed and the annual percentage change (APC) in incidence was calculated by using JoinPoint software.

**Results:**

The crude incidence increased significantly from 23.4/10^5^ in 2000 to 37.4/10^5^ in 2011 for males and from 20.9/10^5^ to 30.5/10^5^ for females. The ASRs of CRC incidence stabilized during the period of 2000–2011 for both males and females. The ages at the onset of CRC for both males and females during 2010–2011 were significantly higher compared with those during 2000–2002 (males: *t* = 1.95, *P* = 0.05; females: *t* = 6.03, *P* < 0.01). For males aged 50–64 years, the CRC incidence increased by 8.50% annually (*P* = 0.04) during 2000–2004 and by 1.68% annually (*P* = 0.03) during 2005–2011. For females aged 65 years and older, the CRC incidence increased by 5.77% annually (*P* = 0.03) during 2000–2004. There were no significant changes for the CRC incidences in males aged 49 and younger and 65 years and older and females aged 64 years and younger during 2000–2004, or for those in all females as well as males aged 49 years and younger and 65 years and older during 2005–2011. The percentage of colon cancer in all CRCs increased significantly for both males and females between the periods of 2000–2002 and 2010–2011. The ASRs of descending colon and sigmoid colon cancer incidences increased significantly for females during 2005–2011 (APC, 5.51% and 1.08%, respectively, both *P* < 0.05).

**Conclusions:**

The crude incidence of CRC increased significantly between 2000 and 2011 because of the aging, whereas the ASRs kept stable. The percentage of colon cancer in all CRCs increased significantly. Further surveillance, research, and intervention are needed to identify the causes of these changes and to reduce the incidence and mortality of CRC.

## Background

Colorectal cancer (CRC) is the third most common cancer in males and the second most common in females worldwide, with the highest incidences found in some developed countries in Oceania (Australia and New Zealand), Europe, and North America [1, 2]. Interestingly, significant international variations have been observed in the distribution of CRC during 1963–2002 [3]. The incidences of CRC are rapidly increasing in several areas between 1963 and 2002, whereas decreasing or stabilizing in the United States, Canada, and New Zealand during 1973–2002 [3].

CRCs are made up of a set of cancers that develop in different anatomic locations of the colon and rectum, including the ascending colon, transverse colon, descending colon, sigmoid colon, and rectum. CRCs had heterogeneous anatomic subsite distribution in terms of CRC incidence, etiology, and clinical and pathologic characteristics [4].

In China, CRC is the third most common cancer [5]. Both the crude incidence and the age-standardized rate (ASR) increased during the period of 2003–2007 at urban areas of China [6, 7]. A previous study reported that the percentage of rectal cancer decreased, whereas the percentage of proximal colon cancer increased during the past 20 years [8]. The present study aimed to describe CRC incidence and age distribution by CRC subsite and to analyze the CRC incidence trends between 2000 and 2011 by analyzing the data from the Guangzhou Cancer Registry.

## Methods

### Data source

All cancer cases were collected from the Guangzhou Cancer Registry. Patient data, including clinicopathologic characteristics, were reported to the Guangzhou Cancer Registry System. Non-residents were excluded. Continuous data between 2000 and 2004 were available for urban areas at Guangzhou, which covered six districts before the adjustment of the administrative division in 2005. These six districts were Yuexiu, Dongshan, Liwan, Fangcun, Haizhu, and Baiyun. The data between 2005 and 2011 were available for the 12 districts in Guangzhou, which were defined for the adjustment of the administrative division in 2005. New Yuexiu district includes old Yuexiu and Dongshan districts, and some streets of old Baiyun and Tianhe districts. New Liwan district includes old Liwan and Fangcun districts. Old Panyu district was divided into Panyu and Nansha districts. Luogang is a new district including some streets of Baiyun, Huangpu, Tianhe, and Zengcheng districts. Population data for Guangzhou was obtained from the Statistics Bureau of Guangzhou.

All cancers of the colon and rectum identified as codes C18-C20 from the 10th revision of the International Classification of Diseases (ICD-10) were included for analysis. For subsite analysis, colon cancers were classified as ascending colon, transverse colon, descending colon, and sigmoid colon cancers. Rectal cancer included recto-sigmoid junction cancer and rectal cancer.

### Statistical analyses

The crude incidence, age-specific rate, and ASR were calculated annually between 2000 and 2011. The ASR was age-adjusted according to the Segi’s world standard population. The annual percentage changes (APCs) were calculated and trend tests were performed by using the Jointpoint Regression Program 4.0.4, which was downloaded from the website of the National Cancer Institute (NCI, MD, USA). The softwares including IARCcrgTools 2.05 issued by IARC (Lyon, France) and SAS 9.1 software (SAS Institute Inc., NC, USA) were used for data checking and statistical analyses.

## Results

### Crude incidence

CRC incidence in males increased from 23.4/10^5^ in 2000, accounting for 9.19% of all new cancer cases and ranking the fifth of all cancer sites, to 37.4/10^5^ in 2011, accounting for 13.28% of all new cancer cases, and ranking the third of all cancer sites, respectively. In females, CRC incidence increased from 20.9/10^5^ in 2000, accounting for 10.76% of all new cancer cases and ranking the fourth of all cancer sites, to 30.5/10^5^ in 2011, accounting for 13.12% of all new cancer cases and ranking the third of all cancer sites, respectively (Table 1).

**Table 1:** Crude incidences and ASRs of colorectal cancer incidence between 2000 and 2011 in Guangzhou

Year	Males				Females				All			
	N	Population	Crude incidence(1/10^5^)	ASR (1/10^5^)	N	Population	Crude incidence (1/10^5^)	ASR (1/10^5^)	N	Population	Crude incidence (1/10^5^)	ASR (1/10^5^)
2000	427	1,824,618	23.4	27.2	362	1,736,192	20.9	16.8	789	3,560,810	22.2	20.5
2001	468	1,874,747	25.0	26.3	379	1,738,975	21.8	19.2	847	3,613,722	23.4	22.1
2002	462	1,890,228	24.4	22.9	386	1,760,021	21.9	17.8	848	3,650,249	23.2	20.0
2003	554	1,900,769	29.1	31.3	423	1,772,209	23.9	20.0	977	3,672,978	26.6	24.7
2004	579	1,879,844	30.8	26.6	459	1,793,754	25.6	19.2	1,038	3,673,598	28.3	22.6
2005	1,343	3,791,319	35.4	29.6	1,042	3,616,274	28.8	20.9	2,385	7,407,593	32.2	25.0
2006	1,274	3,842,030	33.2	26.8	1,039	3,678,044	28.2	20.3	2,313	7,520,074	30.8	23.3
2007	1,405	3,915,761	35.9	28.4	1,096	3,741,759	29.3	20.4	2,501	7,657,520	32.7	24.2
2008	1,539	3,947,724	39.0	30.5	1,178	3,804,461	31.0	21.3	2,717	7,752,185	35.0	25.7
2009	1,475	3,995,986	36.9	28.5	1,207	3,864,080	31.2	20.8	2,682	7,860,066	34.1	24.5
2010	1,449	4,051,193	35.8	26.7	1,122	3,928,858	28.6	19.2	2,571	7,980,051	32.2	22.7
2011	1,539	4,116,884	37.4	27.0	1,225	4,012,543	30.5	19.7	2,764	8,129,427	34.0	23.2
Total	12,514	37,031,103	33.8	27.6	9,918	35,447,170	28.0	19.9	22,432	72,478,273	30.9	23.5

*ASRs* age-standardized rates according to the Segi’s world standard population (per 100,000 persons); *N* number of cases.

### Incidence trends

**Figure 1:**
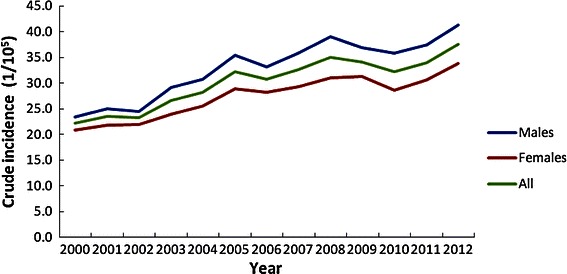
The crude incidence trends of colorectal cancer for both sexes between 2000 and 2011 in Guangzhou.

**Figure 2:**
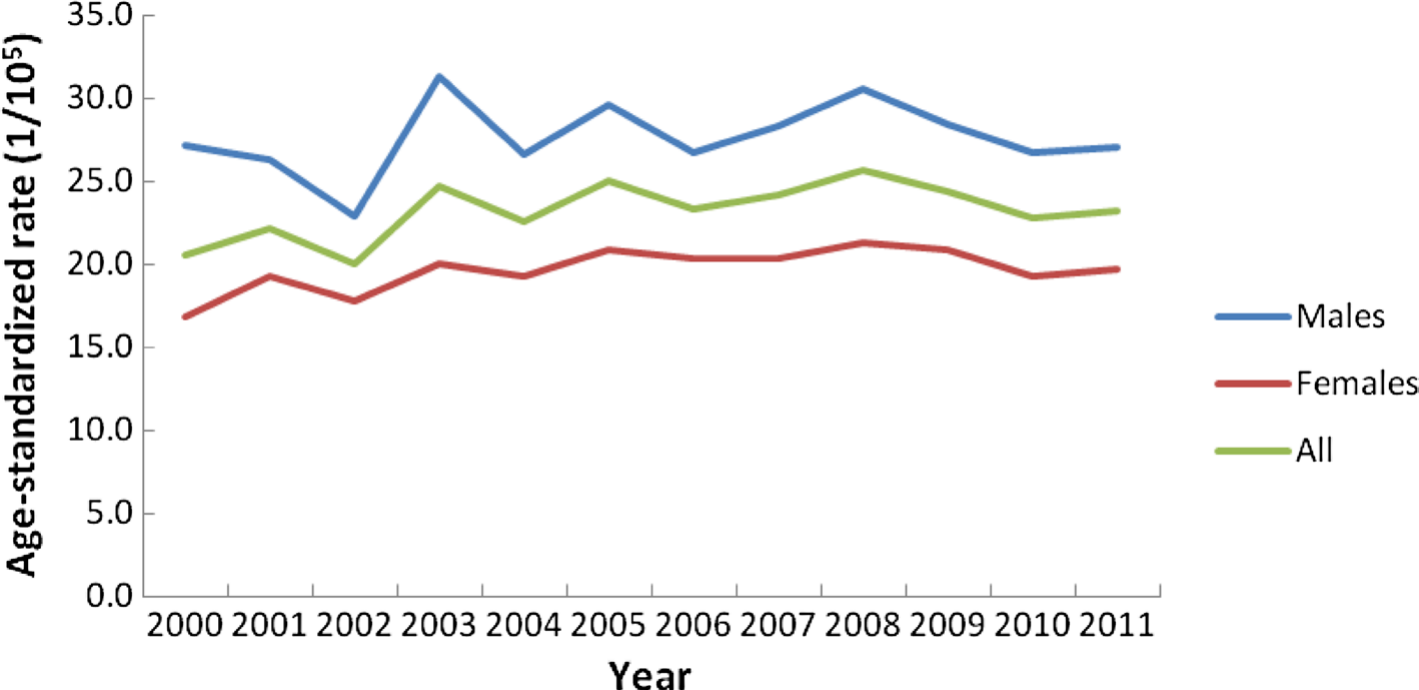
The age-standardized rate trends of colorectal cancer incidences for both sexes between 2000 and 2011 in Guangzhou.

The APCs of CRC crude incidence for males and females were 7.44% (*P* = 0.02) and 5.21% (*P* = 0.01) during 2000–2004 and 1.18% (*P* = 0.25) and 0.93% (*P* = 0.28) during 2005–2011, respectively. The ASRs of CRC incidence stabilized during the period of 2000–2011 for both males and females. The APCs of ASR of CRC incidence for males and females were 1.43% (*P* = 0.75) and 3.06% (*P* = 0.20) during 2000–2004 and −1.02% (*P* = 0.34) and −0.97% (*P* = 0.17) during 2005–2011, respectively (Table 2; Figures. 1, 2).

**Table 2:** APC of colorectal cancer incidence in different age groups during 2000–2011 in Guangzhou

Age group	During 2000–2004		During 2005–2011	
	APC (%)	*P* value	APC (%)	*P* value
All (years)				
0–49	2.49	0.48	−3.07	<0.01
50–64	6.48	0.06	0.49	0.64
≥65	2.23	0.62	0.11	0.93
Crude incidence	6.43	0.01	1.05	0.04
ASR	3.08	0.32	−0.99	0.27
Males (years)				
0–49	7.25	0.18	−3.45	0.06
50–64	8.50	0.04	1.68	0.03
≥65	−1.76	0.77	−0.39	0.70
Crude incidence	7.44	0.02	1.18	0.25
ASR	1.43	0.75	−1.02	0.34
Females (years)				
0–49	−2.07	0.64	−2.56	0.21
50–64	4.13	0.14	−1.12	0.29
≥65	5.77	0.03	0.77	0.61
Crude incidence	5.21	0.01	0.93	0.28
ASR	3.06	0.20	−0.97	0.17

*APC* annual percentage change. Other abbreviation as in Table 1.

### Age distribution

**Figure 3:**
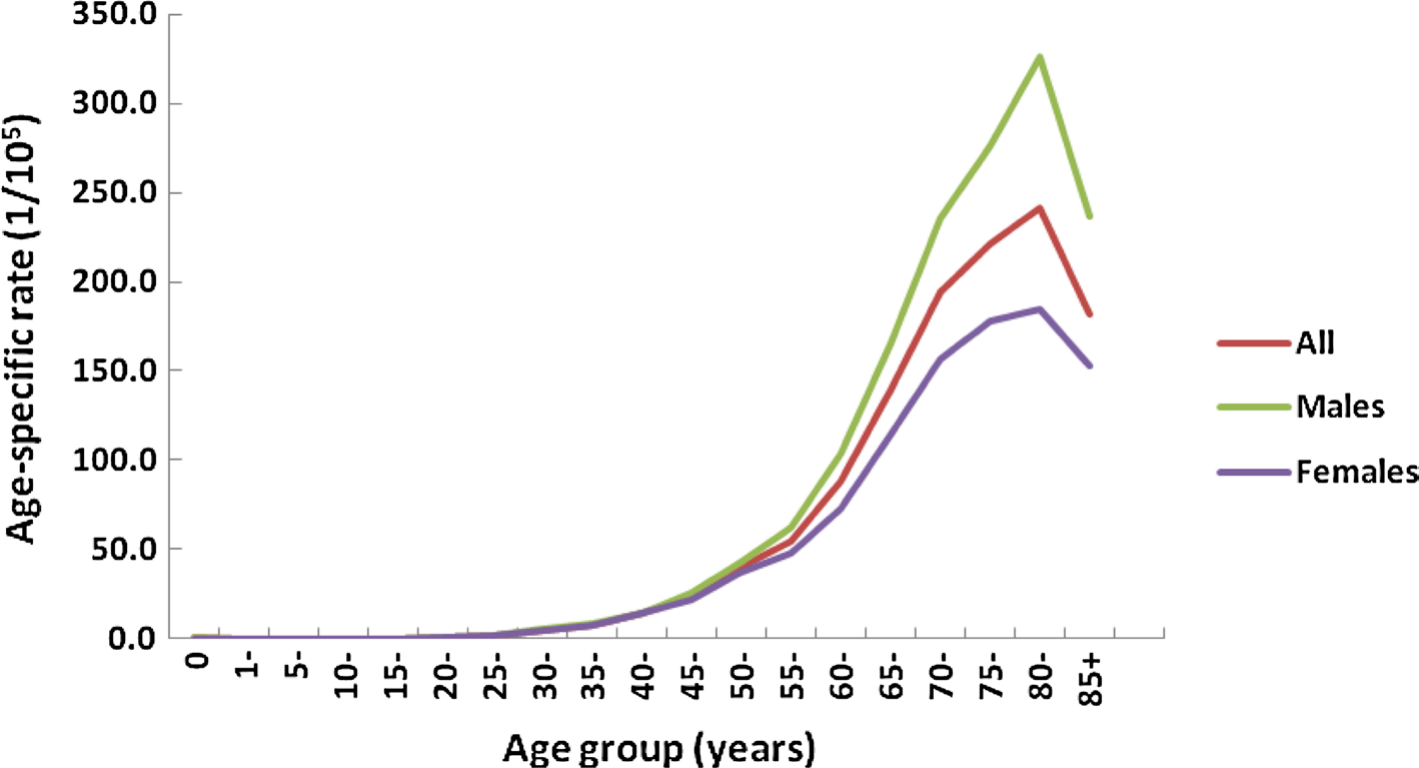
The age-specific rate trends of colorectal cancer incidences for both sexes between 2000 and 2011 in Guangzhou.

Figure 3 presents age-specific rates of CRC incidence during 2000–2011 in Guangzhou. The age-specific rates were relatively low in populations younger than 40 years for both males and females. There was a dramatic increasing trend in the CRC incidence after 40 years of age, which peaked at 80 years of age, and then declined after 85 years of age. The age-specific rates were higher in males than in females for all age groups. The ages at the onset of CRC for both males and females during 2000–2002 were significantly younger than those during 2010–2011 (males: 64.6 ± 0.4 years vs. 65.5 ± 0.2 years, *t* = 1.95, *P* = 0.05; females: 62.8 ± 0.4 years vs. 65.8 ± 0.3 years, *t* = 6.03, *P* < 0.01).

Table 2 presents the APCs of CRC incidence during 2000–2011 for different age groups. The CRC incidence increased by 8.50% (*P* = 0.04) annually during 2000–2004 and 1.68% (*P* = 0.03) during 2005–2011 for males aged from 50 to 64 years and increased by 5.77% (*P* = 0.03) during 2000–2004 and 0.77% (*P* = 0.61) during 2005–2011 for females aged 65 or older.

### Subsite distribution

The percentages of ascending colon, transverse colon, descending colon, sigmoid colon, and rectal cancer cases during 2000–2011 were 11.62%, 7.22%, 4.90%, 20.91%, and 41.75% in males and 14.63%, 8.23%, 4.80%, 20.24%, and 36.96% in females, respectively. The percentage of cases classified as not otherwise specified (NOS) was 13.60% for males and 15.14% for females.

**Figure 4:**
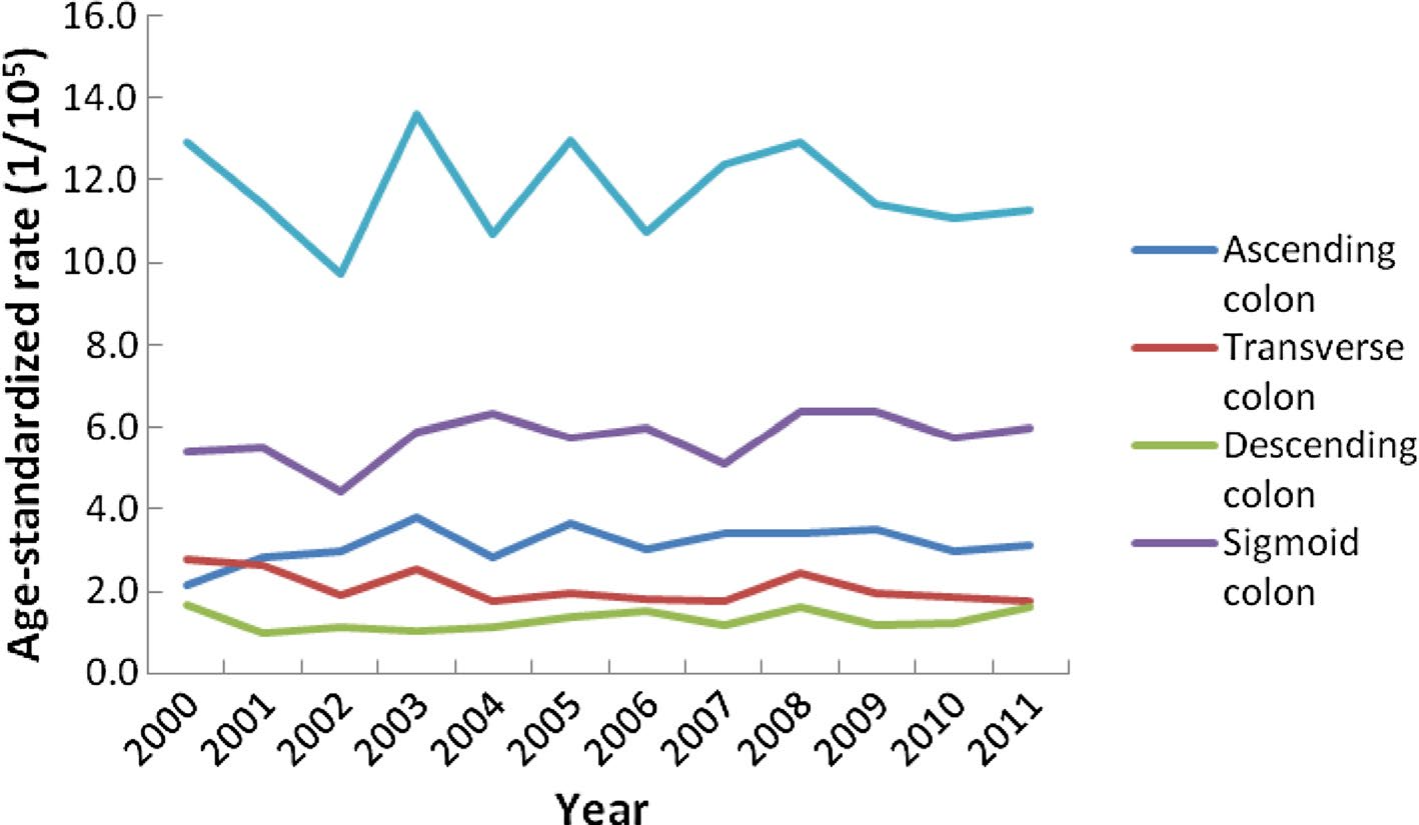
The age-standardized rate trends of colorectal cancer incidence by subsite in males between 2000 and 2011 in Guangzhou.

**Figure 5:**
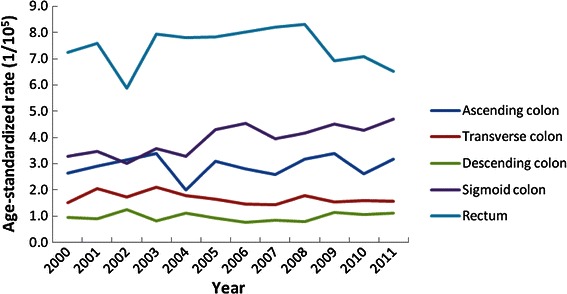
The age-standardized rate trends of colorectal cancer incidence by subsite in females between 2000 and 2011 in Guangzhou.

The percentage of colon cancer in all CRCs increased significantly in both males and females during 2010–2011 compared with those during 2000–2002 (males: 59.34% vs. 55.49%, *P* < 0.05; females: 65.66% vs. 61.85%, *P* < 0.05). Figures 4 and 5 show the trends in ASRs of CRC incidence by subsite in males and females, respectively. There were significant increases in the ASRs of descending colon cancer incidence and sigmoid colon cancer incidence for females during 2005–2011 (APC, 5.51% and 1.08%, respectively, both *P* < 0.05).

The crude incidences of descending colon cancer and sigmoid colon cancer increased by 10.00% (*P* < 0.05) and 2.65% (*P* < 0.05), respectively, for males aged 50–64 years from 2005 to 2011. In females aged 65 years and older, there were significant increases in the crude incidences of descending colon cancer and sigmoid colon cancer (APC, 5.04% and 4.34%, respectively, both *P* < 0.05). In contrast, the crude incidence of rectal colon cancer decreased significantly by 3.42% (*P* < 0.05) in females from 2005 to 2011 (Table 3).

**Table 3:** APC of colorectal cancer incidence in different age groups by subsite between 2000 and 2011 in Guangzhou

Site	APC during 2000–2004 (%)				APC during 2005–2011 (%)			
	All	0–49 years	50–64 years	≥65 years	All	0–49 years	50–64 years	≥65 years
All								
Ascending colon	2.25	−9.25	15.08	1.33	−0.44	−6.16	−0.21	2.19
Transverse colon	−1.95	5.29	2.21	−5.09	−0.4	−1.75	−0.25	1.42
Descending colon	−1.02	−7.08	0.61	2.36	2.79	5.31	7.81*	1.77
Sigmoid colon	4.19	11.11*	3.93	2.8	0.97	–2.98	1.07	3.57*
Rectum	0.68	3.01	5.00^*^	−1.83	−2.43	–5.02^*^	−0.54	−1.81
Males								
Ascending colon	7.5	−5.36	25.89	3.04	−1.7	−4.72	−2.74	1.16
Transverse colon	−8.85	10.29	−4.98	−12.93*	−0.82	–2.98	1.89	−0.47
Descending colon	−7.13	2.05	3.05	−11.9	0.77	3.31	10.00*	−0.54
Sigmoid colon	4.61	16.79	5.58	4.1	0.87	−4.99	2.65*	3.01
Rectum	−1.96	5.72	7.48	−7.16	−1.72	−5.42	0.91	−1.26
Females								
Ascending colon	−2.81	−14.3	6.93	–0.34	−0.87	−7.14	2.84	3.1
Transverse colon	3.41	−0.79	8.9	2.57	−0.18	0.17	−2.96*	3.42
Descending colon	2.71	−12.39	−0.46	17.38	5.51*	7.46	5.46	5.04*
Sigmoid colon	0.41	5.6	1.57	−1.11	1.08*	−1.04	−0.8	4.34*
Rectum	2.13	0.53	2.29	4.3	−3.42*	−4.56*	−2.99	−2.55

*APC* annual percentage change. * APC is significantly different from zero (two-sided *P* < 0.05).

## Discussion

CRC is one of the most common cancers in Guangzhou. The ASRs of CRC incidence in Guangzhou during 2000–2011 were higher than the average ASR for urban China in 2010 [9]. Guangzhou has been one of the most economically developed cities in China for the last 30 years. Therefore, the impact of risk factors such as obesity, unhealthy diet, and lack of physical activity on CRC may be more significant there than those for developing cities [10–13].

The present study showed that the crude incidence of CRC increased significantly between 2000 and 2011 in Guangzhou, whereas the ASR stabilized. The crude incidence increased more than the ASR. These findings were consistent with results from the National Central Cancer Registry Database between 1998 and 2007 [14]. However, these results were not entirely consistent with those of a previous study in Shanghai and Dalian city, in which both the crude incidence and the ASRs of CRC incidence increased during 1973–2000 and during 1991–2010 [15, 16]. A possible reason for this difference is that most cases of CRC developed slowly over several years and were influenced by the lifestyle. Therefore, long-term surveillance is needed to identify this trend. In a decade, the aging population may be the most important reason for the crude incidence increase and the delay in the age of onset, like in many other urban areas in China [17].

It is of concern that the greatest increase in the CRC incidence occurred in males aged 50–64 years and in females aged 65 years and older. The percentage of colon cancer in all CRCs increased significantly in both males and females, whereas the percentage of rectal cancer decreased. Specifically, the ASRs of descending and sigmoid colon cancer incidences in females increased significantly, whereas the ASR of rectal cancer incidence decreased significantly. These results were similar to those of a previous study in Osaka, Japan [18]. A gradual left-to-right shift of CRC incidence was observed all over the world [8, 19].

Many studies have shown that population-based colorectal screening, including fetal occult blood testing and colonoscopy, is effective for early diagnosis of CRC and therefore may reduce CRC incidence and mortality because of removal of precancerous lesions, but organized screening programs are yet to be implemented in most countries [1, 20–22]. Therefore, further studies are needed for a reasonable screening method.

The present study has some notable limitations. First, the data included a substantial number of colon cancer cases without specific subsite information. Second, the percentage of colon NOS cases in all CRC cases was 14.28%. Third, a period of 12 years is not sufficient for analyzing trends of CRC incidence, especially because there was a great change in Guangzhou district distribution in 2005. Therefore, the CRC incidence trend for the period of 2000–2004 did not quite match that for the period of 2005–2011. Cancer registration is a long-term continuous task based on a stable population. Therefore, continued cancer registration is needed in Guangzhou, and further studies on factors related to CRC incidence trend and intervention for CRC are also warranted.

## Conclusions

The crude incidence of CRC increased significantly in Guangzhou between 2000 and 2011 because of the aging, whereas the ASRs kept stabilizing. The percentage of colon cancer in all CRCs increased significantly. Further surveillance, research, and intervention are needed to identify the causes of these changes and to reduce the incidence and mortality of CRC in different populations and age groups.

## References

[1] Jemal A, Bray F, Center MM, Ferlay J, Ward E, Forman D (2011). Global cancer statistics. CA Cancer J Clin.

[2] Center MM, Jemal A, Smith RA, Ward E (2009). Worldwide variations in colorectal cancer. CA Cancer J Clin.

[3] Center MM, Jemal A, Ward E (2009). International trends in colorectal cancer incidence rates. Cancer Epidemiol Biomarkers Prev.

[4] Minoo P, Zlobec I, Peterson M, Terracciano L, Lugli A (2010). Characterization of rectal, proximal and distal colon cancers based on clinicopathological, molecular and protein profiles. Int J Oncol.

[5] Chen W, Zheng R, Zhang S, Zhao P, Li G, Wu L (2013). The incidences and mortalities of major cancers in China, 2009. Chin J Cancer..

[6] Zhao P, Chen WQ, Kong LZ (2012). Cancer incidence and mortality in China (2003-2007).

[7] Chen WQ, Zheng RS, Zhang SW, Zeng HM, Zou XN (2014). The incidences and mortalities of major cancers in China, 2010. Chin J Cancer.

[8] Xu AG, Jiang B, Zhong XH, Yu ZJ, Liu JH (2006). The trend of clinical charac teristics of colorectal cancer during the past 20 years in Guangdong province. Zhonghua Yi Xue Za Zhi.

[9] Zheng ZX, Zheng RS, Zhang SW, Chen WQ (2014). Colorectal cancer incidence and mortality in China, 2010. Asian Pac J Cancer Prev.

[10] Theodoratou E, Montazeri Z, Hawken S, Allum GC, Gong J, Tait V (2012). Systematic meta-analyses and field synopsis of genetic association studies in colorectal cancer. J Natl Cancer Inst.

[11] Bardou M, Barkun AN, Martel M (2013). Obesity and colorectal cancer. Gut.

[12] Cappellani A, Zanghì A, Di Vita M, Cavallaro A, Piccolo G, Veroux P (2013). Strong correlation between diet and development of colorectal cancer. Front Biosci Landmark Ed.

[13] Spence RR, Heesch KC, Brown WJ (2009). A systematic review of the association between physical activity and colorectal cancer risk. Scand J Med Sci Sports.

[14] Dai Z, Zheng RS, Zou XN, Zhang SW, Zeng HM, Li N (2012). Analysis and prediction of colorectal cancer incidence trend in China. Zhonghua Yu Fang Yi Xue Za Zhi..

[15] Zhang LM, Shao SL (2013). An analysis on epidemic trend of colorectal cancer from 1991 to 2010 in Dalian city. Zhongguo Zhong Liu.

[16] Gao YT, Lu W (2007). Cancer incidence, mortality and survival rates in urban Shanghai (1973–2000).

[17] Li HL, Gao YT, Zheng Y, Zhang W, Gao LF, Xu B (2009). Incidence trends of colorectal cancer in urban Shanghai, 1973–2005. Zhonghua Yu Fang Yi Xue Za Zhi..

[18] Toyoda Y, Nakayama T, Ito Y, Ioka A, Tsukuma H (2009). Trends in colorectal cancer incidence by subsite in Osaka, Japan. Jpn J Clin Oncol.

[19] Caldarella A, Crocetti E, Messerini L, Paci E (2013). Trends in colorectal incidence by anatomic subsite from 1985 to 2005: a population-based study. Int J Colorectal Dis.

[20] Shaukat A, Mongin SJ, Geisser MS, Lederle FA, Bond JH, Mandel JS (2013). Long-term mortality after screening for colorectal cancer. N Engl J Med.

[21] Brenner H, Kloor M, Pox CP (2014). Colorectal cancer. Lancet.

[22] Brenner H, Stock C, Hoffmeister M (2014). Effect of screening sigmoidoscopy and screening colonoscopy on colorectal cancer incidence and mortality: systematic review and meta-analysis of randomised controlled trials and observational studies. BMJ.

